# Differential Activity of the Extracellular Phenoloxidases in Different Strains of the Phytopathogenic Fungus, *Microdochium nivale*

**DOI:** 10.3390/jof8090918

**Published:** 2022-08-29

**Authors:** Elena Vetchinkina, Azat Meshcherov, Vladimir Gorshkov

**Affiliations:** 1 Institute of Biochemistry and Physiology of Plants and Microorganisms, Saratov Scientific Centre of the Russian Academy of Sciences (IBPPM RAS), 13 Prospekt Entuziastov, 410049 Saratov, Russia; 2 Kazan Institute of Biochemistry and Biophysics, FRC Kazan Scientific Center of Russian Academy of Sciences, 2/31 Lobachevskogo, 420111 Kazan, Russia; 3 Kazan Federal University, 420008 Kazan, Russia

**Keywords:** phenotypic diversity of phytopathogenic fungi, phenoloxidases, extracellular enzymes of fungi, plant infectious diseases, *Microdochium nivale*

## Abstract

To cause plant diseases, phytopathogenic fungi use numerous extracellular enzymes, among which, the phenoloxidases (POs) seem underestimated for the pathogens of non-woody plants. Our study aimed to (1) compare extracellular PO activities (lignin peroxidase, Mn peroxidase, laccase, and tyrosinase) in differentially virulent strains (inhabiting winter rye in a single field) of the phytopathogenic species, *Microdochium nivale*; (2) check whether these activities are responsive to host plant metabolites; and (3) search for correlations between the activities, lignin-decomposing capacity, and virulence. All strains displayed all four enzymatic activities, but their levels and dynamics depended on the particular strain. The activities displayed the hallmarks of co-regulation and responsiveness to the host plant extract. No relationships between the virulence of strains and levels of their extracellular PO activities or lignin-degrading capacity were revealed. We consider that different strains may rely on different POs for plant colonization, and that different POs contribute to the “uniqueness” of the enzymatic cocktails that are delivered into host plant tissues by different virulent strains of *M. nivale*. Our study supports the hypothesis of the differential behavior of closely related *M. nivale* strains, and discusses an important role of POs in the interactions of phytopathogens with herbaceous plants.

## 1. Introduction

Plant infectious diseases caused by phytopathogenic fungi are often coupled with the destruction of plant tissues by fungal enzymes. Most of these enzymes destroy plant cell wall (PCW) polymers—polysaccharides (cellulose, cross-linking glycans, and pectins) and different proteins. Due to this, extracellular fungal enzymes involved in polysaccharide and protein depolymerization have been extensively studied [1,2]. In addition to polysaccharides and proteins, the fortification of the PCW is also provided by phenolic compounds, most of which are represented in the PCW by lignin and phenolic acids attached to polysaccharides [3]. The substitution with phenolic acids enables polysaccharides to form strong linkages with each other, as well as with lignin and aromatic amino-acid-containing proteins, leading to the emergence of a polymeric network that is difficult to degrade by pathogen enzymes [4]. Therefore, the enzymes that catalyze the destruction of phenolic compounds—phenoloxidases (POs)—seem to be an important tool of phytopathogens. Moreover, in addition to providing PCW degradation, POs may have the potential to deactivate some plant defense compounds, phytoalexins, many of which are of phenolic nature, and reduce oxidative stress by acting as antioxidant enzymes [5]. However, in phytopathogens, at least in those that cause diseases in non-woody plants, POs are poorly characterized by only a few examples [6,7,8,9,10].

POs are extensively studied in basidiomycetes (*Basidiomycota*), especially xylotrophic ones that cause wood decay. The ligninolytic enzyme complex of basidiomycetes consists mostly of oxidoreductases that catalyze lignin degradation: lignin peroxidases (ligninases, EC 1.11.1.14), manganese (Mn)-dependent peroxidases (Mn peroxidases, EC 1.11.1.13), and polyphenol oxidases (laccases, EC 1.10.3.2, and tyrosinase, EC 1.14.18.1) [11,12,13,14]. The POs of fungi from *Ascomycota* have been significantly less studied. By screening 610 strains of 16 species isolated from soil, compost, and decayed wood, more than 50 ascomycete strains have been shown to produce extracellular POs [15]. *Aspergillus flavus* and *Aspergillus nidulans*, which belong to the *Eurotiomycetes* class of *Ascomycota*, have been shown to be able to degrade alkali lignin and exhibit Mn peroxidase, lignin peroxidase, and laccase activities [16]. In the representative of the class, *Leotiomycetes*, *Botrytis cinerea*, a well-known causal agent of gray rot of different plants, laccases and peroxidases act as quenchers of free radicals and reactive oxygen species (ROS) during the plant defense response (hypersensitive response) [17].

Laccases were revealed in many species from the *Dothideomycetes* class: *Botryosphaeria rhodina* [18], *Botryosphaeria kuwatsukai* [19], *Clavariopsis aquatic* [20], *Coniothyrium* sp. [21], *Hortaea acidophila* [22,23], *Phoma* sp. [24], and *Paraconiothyrium variabile* [25]. In addition to laccases, *Paraconiothyrium variabile*, a species found in wood-damaged stems and vines of different plants (*Actinidia chinensis*, *Actinidia deliciosa*, *Laurus nobilis*, *Prunus persica*, and *Prunus salicina*), has been demonstrated to produce Mn peroxidases and lignin peroxidases, and to decompose model and native lignin, guaiacol, polymeric dyes (phenol red and aniline blue), and antracen derivatives [25]. The knockout of one of the laccase-encoding genes (BkLiP7) in *Botryosphaeria kuwatsukai*, a phytopathogenic fungus that infects a wide range of woody plants, had a negative effect on the virulence of this species [19].

Within the class, *Sordariomycetes*, laccases were revealed in the species of eight families: *Chaetomiaceae*—*Chaetomium thermophilium* [26], *Melanocarpus albomyces* [27,28], *Hypocreaceae*—*Trichoderma atroviride* [29,30], *Trichoderma harzianum* [31], *Microascaceae*—*Petriellidium fusoideum* [32], *Magnaporthaceae*—*Gaeumannomyces graminis* [33], *Niessliaceae*—*Monocillium indicum* [34], *Sordariaceae*—*Neurospora crassa* [35], *Xylariaceae*—*Xylaria polymorpha*, *Xylaria hypoxylon* [36,37], and *Glomerellaceae*—*Colletotrichum orbiculare* [10]. In *Coniochaeta ligniaria*, laccase activity has not been detected; however, this fungus displayed lignin peroxidase and Mn peroxidase activities [38]. In *Colletotrichum orbiculare*, a causal agent of anthracnose of plants from the *Cucurbitaceae* family, as well as in many other fungal species, laccases (including LAC2) are involved in melanization that occurs, in particular, during the formation of appressoria necessary for the penetration of plant tissues. The *lac2* mutant strain of *C. orbiculare* was unable to form functional appessoria and invade the leaves of the host plant [10].

The genera from the *Xylariales* order (*class Sordariomycetes*) mostly include xylotrophs, many of which cause wood soft rot [39]. The representatives of this order, *Xylaria polymorpha* and *Xylaria hypoxylon*, have been shown to be able to decompose the lignocellulose substrate and produce extracellular laccases. Herewith, the extracellular activities of lignin peroxidase and Mn peroxidase have not been revealed in these species [36,37]. In addition to xylotrophs, the *Xylariales* order contains the genus, *Microdochium*, which is represented, in particular, by the devastating phytopathogenic species, *Microdochium nivale* (Fr.) Samuels and Hallett, and *M. majus* (Wollenw.) Glynn and S.G. Edwards [40]. This species causes various diseases in non-woody plants, such as cereals and annual or perennial grasses [41,42,43,44,45]. *M. nivale* is one of the causal agents of snow mold disease, which develops under the snow cover in winter cereals/grasses, and often reaches the epiphytotic level. However, *M. nivale*-caused pathogenesis is not strictly coupled with the presence of snow cover, and this species may also cause foot rot, leaf blight, head blight, and other types of diseases throughout the growing period [46,47,48].

High genetic and phenotypic diversity is typical of *M. nivale* species [43,49,50]. In our recent study, we isolated 21 strains of *M. nivale* from a single territory (single field) and a particular crop (winter rye), and showed that these strains differed strongly from each other in terms of virulence and the level of production of different extracellular enzymes (cellulase, xylanase, arabinofuranosidase, pectate lyase, protease, amylase, and invertase) [50]. We also revealed that these strains had extracellular lignin peroxidase activity. However, other POs have not been previously analyzed in *M. nivale*. Therefore, our study aimed to analyze whether *M. nivale*—a pathogen of non-woody plants—produces different types of extracellular POs, to compare extracellular PO activities in the cultures of different strains, to check whether these activities are responsive to the presence of host plant metabolites, and to search for correlations between the levels of enzymatic activities and virulence.

## 2. Materials and Methods

### 2.1. Culture Conditions

Twenty-one strains of *Microdochium nivale* collected at the field of the Federal Research Center “Kazan Scientific Center of the Russian Academy of Sciences”, located in the forest–steppe area of the Volga region, Laishev District, Tatarstan Republic, Russia (Universal Transverse Mercator (UTM) north (N) 55.649 east (E) 49.3083), [50] were used in this study. The strains are referred to as 1–21 throughout the paper; the corresponding accession numbers in the collection are given in Table S1 (Supplementary Materials). The virulence levels of the strains are described in Table S2 (Supplementary Materials).

For the determination of PO activities, all strains were grown submerged in two media: potato sucrose broth (PSB), containing (g/L) potato—200; sucrose—30, pH 6.2, and minimal medium (MM), containing (g/L) D-glucose—3; L-asparagine—0.3; KH_2_PO_4_—2; K_2_HPO_4_—3; MgSO_4_ × 7H_2_O—2.5; FeSO_4_ × 7H_2_O—0.03, pH 5.8, at 20 °C for 30 days in 100-mL flasks with 50 mL of the medium. As the inoculum, 5-mm mycelial plugs cut from the periphery of 10–14-day old cultures grown on 1.5%-agarized PSB or MM media, respectively, were used (1 plug per flask). The enzymatic activities were assayed at 10-, 20-, and 30-days post-inoculation in three biological replicates.

To assess the effect of the host plant extract on the assayed enzymatic activities, *M. nivale* strain 1 was cultured in MM for 10 days. Then, 1/10 (*v*/*v*) of distilled water or 1/10 (*v*/*v*) of water extract of rye leaves was added to the fungal cultures, and the activities were measured prior to and 12, 24, 48, 96, and 168 h afterward in five biological replicates. To obtain the rye extract, 100 g of fresh rye leaves (grown in vermiculite under a 16/8 light/dark period for 14 days) were thoroughly ground in three volumes (*w*/*v*) of distilled water, and the obtained suspension was filtered through the gauze. The remaining debris was again thoroughly ground in two volumes of water and filtered. Two portions of the obtained filtrates were combined and centrifuged (10,000× *g*, room temperature, 10 min). The supernatants were collected, incubated for 10 min at 80 °C, and centrifuged again. Further, the extracts were sterilized through nitrocellulose filters with pores of 0.22 μm in diameter (Corning Incorporated, New York, NY, USA) under sterile conditions, and kept frozen (−20 °C) until use.

### 2.2. Enzymatic Activity Assays

To determine the extracellular lignin peroxidase, Mn peroxidase, laccase, and tyrosinase activities, supernatants of fungal cultures were collected and centrifuged (10,000× *g*, 10 min, at room temperature). The activities were measured spectrophotometrically at 18 °C in 96-well polystyrol microplates on a Spark 10M (“Tecan” Group Ltd., Mannedorf, Switzerland). As a control, an equivalent volume of sterile medium (MM (with and without rye extract) or PSB) was used.

The lignin peroxidase activity was determined by the rate of oxidation of veratric alcohol to veratraldehyde [51]. Here, 140 μL of 2 mM veratryl alcohol (Acros Organics, Fair Lawn, NJ, USA) in 100 mM sodium tartrate buffer (pH 3.0) was mixed with 10 μL of 0.4 mM H_2_O_2_ and 50 μL of the cultural supernatant. The accumulation of the veratraldehyde (ε = 9.3 mM^−1^ cm^−1^) was measured at 310 nm.

The activity of Mn peroxidase was determined by the rate of oxidation of 2,6-dimethoxyphenol (DMOP; Sigma-Aldrich, Saint Louis, MO, USA) to a coerulignone (3,3′,5,5′-tetramethoxy-p,p’-diphenoquinone) [52]. Here, 100 μL 50 mM of sodium tartrate buffer (pH 4.5), containing 0.5 mM DMOP, 0.2 mM MnSO_4_ × 5H_2_O, and 0.1 mM H_2_O_2_, was mixed with 100 μL of the cultural supernatant. The accumulation of the stable cation radical (ε = 27.5 mM^−1^ cm^−1^) was measured at 468 nm.

Laccase activity was determined by the rate of oxidation of 2,2′-azino-bis-(3-ethylbenzothiazoline-6-sulfonic acid) diammonium salt (ABTS; Sigma, Saint Louis, MO, USA) to a stable cation-radical ABTS+ [6]. Here, 100 μL of 0.2 mM ABTS in 50 mM Na-tartrate buffer (pH 4.5) was mixed with 100 μL of the cultural supernatant. The reaction products (ε = 29.3 mM^−1^ cm^−1^) were measured at 436 nm.

Tyrosinase activity was determined by the rate of oxidation of 3-(3,4-dihydroxyphenyl)-l-alanine (L-DOPA; Serva, Heidelberg, Germany) to DOPA-quinone [53]. Here, 100 μL of 2 mM L-DOPA in 50 mM Tris-HCl buffer (pH 7.5) was mixed with 100 μL of the cultural supernatant. The DOPA-quinone (ε = 3.7 mM^−1^ cm^−1^) was measured at 475 nm.

The time course of all reactions was 10 min. The amount of enzyme catalyzing the formation of 1 μmol of the product per min was considered an activity unit and expressed in μmol/min/mg of protein. The protein concentration was assayed by the Bradford method [54].

### 2.3. Electrophoresis in Polyacrylamide Gel and Staining of Phenol Oxidases

The patterns of *M. nivale* extracellular laccases, Mn peroxidases, and tyrosinases were analyzed by electrophoresis under non-denaturing conditions in a 7.5% polyacrylamide gel (PAGE) [55]. For these experiments, cultures of *M. nivale* grown in PSB for 20 days were used. Cultural supernatants of *M. nivale* strains were separated from the mycelium and centrifuged at 10,000× *g* for 20 min. The supernatants were concentrated 20-fold by lyophilization, and 20 µL-samples were analyzed by electrophoresis.

To visualize the proteins with laccase activity after PAGE, the gels were stained with o-dianisidine (Sigma, Saint Louis, MO, USA) [56]. o-Dianisidine was dissolved in 0.5 mL of concentrated acetic acid, and then added to 50 mL of 50 mM sodium tartrate buffer (pH 4.5). Mn-dependent peroxidase activity was visualized using a solution of 50 mM sodium tartrate buffer (pH 4.5) with 2 mM 2,6-dimethoxyphenol, 0.1 mM H_2_O_2_, and 0.2 mM MnSO_4_ [57]. Tyrosinase activity was detected after PAGE by incubating the gels in a 2 mM solution of L-dihydroxyphenylalanine (L-DOPA, Serva, Heidelberg, Germany) in 50 mM of Tris-HCl buffer (pH 7.5) [53].

### 2.4. Lignin Decomposition Assay

The lignin decomposing capacity of *M. nivale* extracellular enzymes was analyzed for the supernatants of fungal cultures grown for 20 days in MM. Supernatants were separated from the mycelium and centrifuged at 10,000× *g* for 20 min. As a substrate for the reactions, nitrated lignin was used. To obtain the nitrated lignin, 1 mL of glacial acetic acid was added dropwise to 5 mg of native lignin from hardwood (Sigma, Saint Louis, MO, USA). The resulting suspension was stirred (120 turns per min, room temperature) and filtered through nitrocellulose filters with pores of 0.22 μm in diameter (Corning Incorporated, New York, NY, USA) to remove undissolved particles. Then, 0.2 mL of concentrated nitric acid was added, and the mixture was stirred for 1 h (120 turns per min, room temperature). After that, 2 mL of distilled water was added, and the mixture was titrated with 1 M NaOH to pH 7.0. The resultant solution (yellow-orange color) was diluted 100 times with 750 mM Tris-HCl buffer (pH 7.4) with 50 mM NaCl and used as a stock solution for the assays [58].

Lignin decomposition assays were carried out in a reaction mixture containing 160 µL of nitrated lignin solution, 30 µL of supernatant sample, and 10 µL of either 40 mM H_2_O_2_ or water; the total volume was 200 µL. The reactions were carried out both in the presence and absence of H_2_O_2_ [58]. Lignin decomposition products were detected spectrophotometrically at 18 °C in 96-well polystyrol microplates on a Spark 10M (“Tecan” Group Ltd., Mannedorf, Switzerland). The measurement of the reaction products was carried out at 310 nm for 20 min, with a measurement interval of 1 min. As a control, 30 μL of MM was added to the reaction mixture instead of a fungal supernatant sample. The obtained values were normalized to the protein concentration.

To compare lignin decomposition intensity by the enzymes of different strains, the approximation of normalized (normalization was performed based on values of extracellular protein content obtained according to the Bradford method) kinetic curves of lignin degradation product accumulation was performed (the general form of the approximated polynomial function was ax^2^ + bx + c, and the determination coefficient was taken at a level > 0.95). For each approximated function, the derivative of the equation was determined. The equation of the derivative was used to calculate the values of the derivatives at the extreme points (1 and 20 min). The lignin decomposition level was expressed as a module of the difference in derivative values at the extreme points.

### 2.5. Statistical Analysis

Data processing and visualization were performed using Microsoft Excel 2010, XLSTAT version 2016.02.28451, and OriginPro 2019b 9.6.5.169. Cluster analysis was based on Ward’s model. The measure of distance in the agglomeration method was the Euclidean distance. Correlation tests were performed using Spearman’s rank correlation coefficient. Statistically significant differences between groups were determined using the *t*-test and the Mann–Whitney U test. Results were considered significant at a probability level of *p* ≤ 0.05.

## 3. Results

### 3.1. The Dynamics of Activities of Extracellular POs in the Cultures of Different M. nivale Strains

To check whether *M. nivale* produces different types of extracellular POs and, if so, to compare the levels of their activities in the cultures of different strains, extracellular lignin peroxidase (LP), Mn peroxidase (MP), laccase (Lac), and tyrosinase (Tyr) activities were measured. Since the activity of these enzymes depends on culture conditions [59], we used two different media: minimal medium (MM) and potato sucrose broth (PSB). Activities were measured after 10, 20, and 30 days of cultivation. All 21 strains under both conditions at all analyzed time points displayed the assayed extracellular enzymatic activities, but the levels and dynamics of the activities depended on the particular strain and culture medium.

**Figure 1:**
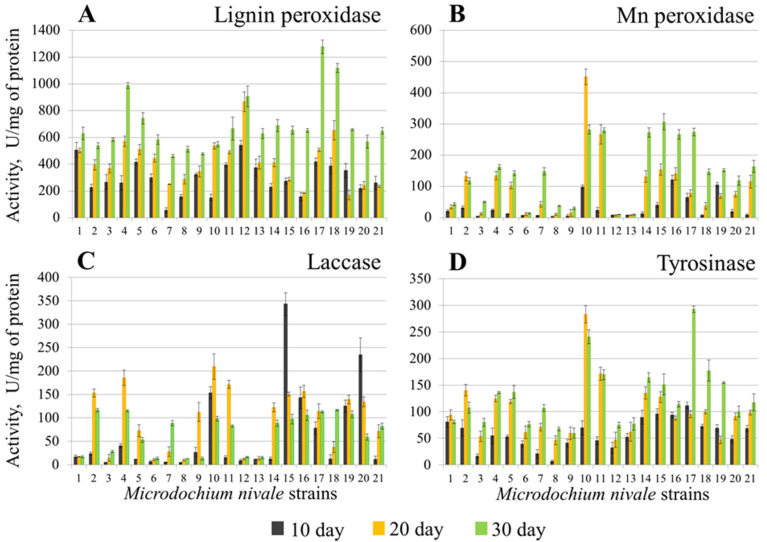
Extracellular lignin peroxidase (**A**), Mn peroxidase (**B**), laccase (**C**), and tyrosinase (**D**) activities of the *Microdochium nivale* strains grown in potato sucrose broth medium. Black columns —10 days of cultivation, orange columns—20 days of cultivation, green columns—30 days of cultivation. The presented values are averages ± SD of three biological replicates.

When *M. nivale* strains were cultured in PSB, the levels of extracellular LP activity varied in different strains in the range of 57–542, 170–867, and 460–1278 U/mg of protein (for 10, 20, and 30 days of incubation, respectively) (Figure 1A), extracellular MP activity—3–121, 7–451, and 10–306 U/mg of protein (for 10, 20, and 30 days of incubation, respectively) (Figure 1B), extracellular Lac activity—3–342, 9–209, and 13–116 U/mg of protein (for 10, 20, and 30 days of incubation, respectively) (Figure 1C), and extracellular Tyr activity—6–111, 46–283, and 59–292 U/mg of protein (for 10, 20, and 30 days of incubation, respectively) (Figure 1D). In most cases, the assayed enzymatic activities increased with the cultivation period under the conditions of PSB. However, in some cases, the levels of enzymatic activities reached their maximum by the 20th day of cultivation (Figure 1). In the cultures of two strains (15 and 20), the extracellular Lac activity was at its maximal level on the 10th day of incubation, and then gradually decreased.

**Figure 2:**
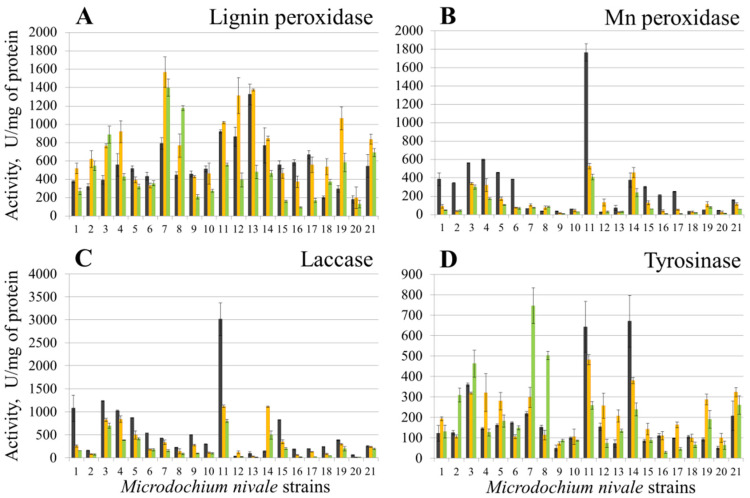
Extracellular lignin peroxidase (**A**), Mn peroxidase (**B**), laccase (**C**), and tyrosinase (**D**) activities of the *Microdochium nivale* strains grown in minimal medium. Black columns —10 days of cultivation, orange columns—20 days of cultivation, green columns—30 days of cultivation. The presented values are averages ± SD of three biological replicates.

The cultivation of *M. nivale* strains in MM resulted in a different pattern of the assayed activities compared to cultures grown in PSB (Figure 2). For example, strains 17 and 18 displayed the highest activity of LP compared to other strains in PSB, whereas the LP activity of these strains was low in MM. Contrarily, strain 7 had the highest LP activity when cultured in MM, whereas its LP activity was one of the lowest among other strains under the conditions of PSB (Figure 2). When strains were cultured in MM, the levels of extracellular LP activities varied in different strains in the range of 183–1326, 200–1567, and 94–1399 U/mg of protein (for 10, 20, and 30 days of incubation, respectively) (Figure 2A), extracellular MP activities—28–1765, 16–528, and 8–408 U/mg of protein (for 10, 20, and 30 days of incubation, respectively) (Figure 2B), extracellular Lac activities—30–3016, 12–1118, and 10–800 U/mg of protein (for 10, 20, and 30 days of incubation, respectively) (Figure 2C), and extracellular Tyr activities—49–670, 71–481, and 28–745 U/mg of protein (for 10, 20, and 30 days of incubation, respectively) (Figure 2D).

In MM, seven and twelve strains displayed maximal extracellular LP activity on the 10th and 20th days of cultivation, respectively (Figure 2A), whereas in PSB, maximal LP activity for all 21 strains was observed on the 30th day of cultivation (Figure 1A). Extracellular MP activity of more than half of the strains cultured in MM reached its maximum on the 10th day of cultivation, and the highest level of Lac activity was also observed on the 10th day of cultivation for 19 of 21 strains (Figure 2B,C). Strain 11 displayed almost three times higher MP and Lac activities compared to other strains under the conditions of MM. Most of the strains (eleven) displayed maximal extracellular Tyr activity on the 20th day of cultivation. The highest Tyr activity was observed for strains 7 (30 days), as well as 11 and 14 (10 days) in MM (Figure 2D).

**Figure 3:**
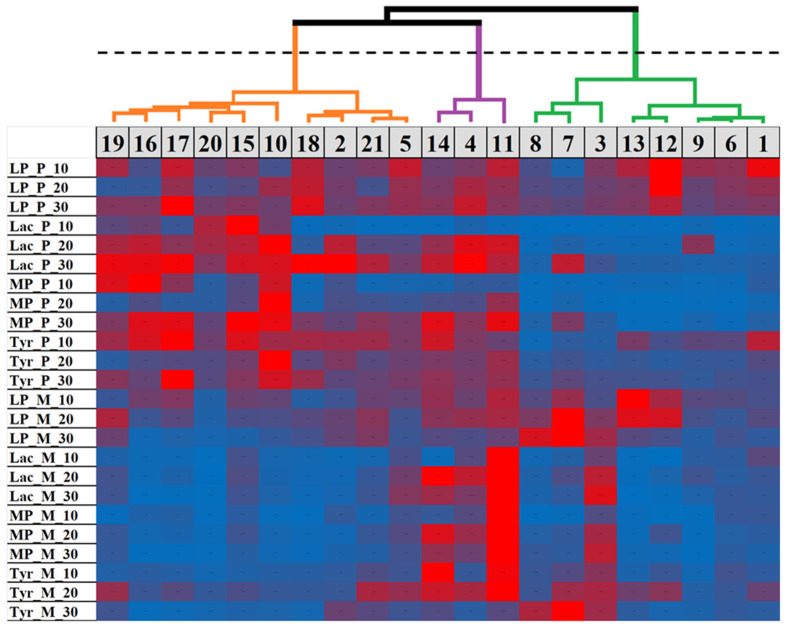
The Hierarchical Clustering Dendrogram combined with a heat map showing the relative patterns of extracellular lignin peroxidase (LP), Mn peroxidase (MP), laccase (Lac), and tyrosinase (Tyr) activities of different *Microdochium nivale* strains, designated by numbers on a gray background. Strains were grown in potato sucrose broth medium (P) or minimal medium (M) for ten (10), twenty (20), or thirty (30) days. The colors correspond to the level of activity (red—maximum and blue—minimum) relative to the activity levels of all analyzed strains. The values (colors) are normalized in relation to a given activity of different strains, but not in relation to different enzymatic activities. The heat map was made using Microsoft Excel 2010 and XLSTAT 2016.02.28451. Clustering was performed using Ward’s method; the proximity between objects was measured by the criteria of dissimilarity based on Euclidian distance. The dotted line shows the cut-off for the depth of analysis (three clusters).

Thus, different *M. nivale* strains display different levels of PO activities both between each other under similar culture conditions, and within a particular strain under different conditions. To reveal the groups with the most similar/different *M. nivale* strains in terms of the pattern of PO activities, a cluster analysis of strains was performed based on the levels of all assayed activities at all three time points under both culture conditions. Three clusters were distinguished (Figure 3). The first cluster included the strains (2, 5, 10, and 15–21) that, on average, displayed relatively high enzymatic activities when cultured in PSB, and relatively low activities when cultured in MM. The strains of the second cluster (4, 11, and 14) had, on average, relatively high PO activities under both assayed conditions. The third cluster included the strains (1, 3, 6–9, 12, and 13) that, on average, had low PO activities under both assayed conditions (Figure 3).

**Figure 4:**
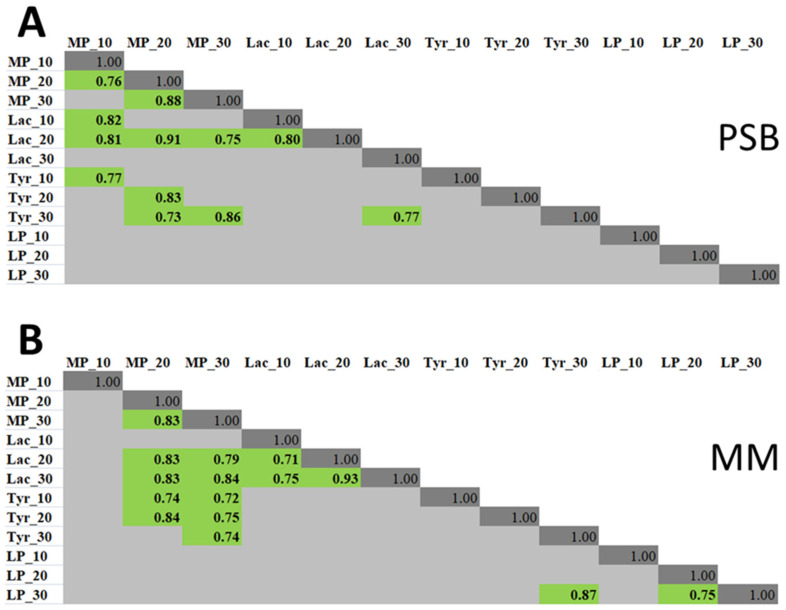
The correlation matrix of the levels of extracellular lignin peroxidase (LP), Mn peroxidase (MP), laccase (Lac), and tyrosinase (Tyr) activities of different *Microdochium nivale* strains grown in (**A**) potato sucrose broth medium (PSB) or (**B**) minimal medium (MM) for ten (10), twenty (20), or thirty (30) days. The presented values are Spearman correlation coefficients. Tests in which the correlations were insignificant (*t*-test with Holm–Bonferroni adjustment, *p*-value < 0.05) are not shown.

To check whether co-regulation of the assayed enzymatic activities may occur, the correlations between different activities were analyzed. Under both conditions (PSB and MM), we found strong correlations between the activities of MP and Lac, as well as MP and Tyr (Figure 4). No correlations between LP activity and the activities of other POs were revealed except in one case: for the PSB culture conditions, on the 30th day of cultivation, the level of LP activity correlated with the level of Tyr activity.

### 3.2. The Analysis of M. nivale POs by Native Gel-Eletrophoresis

**Figure 5:**
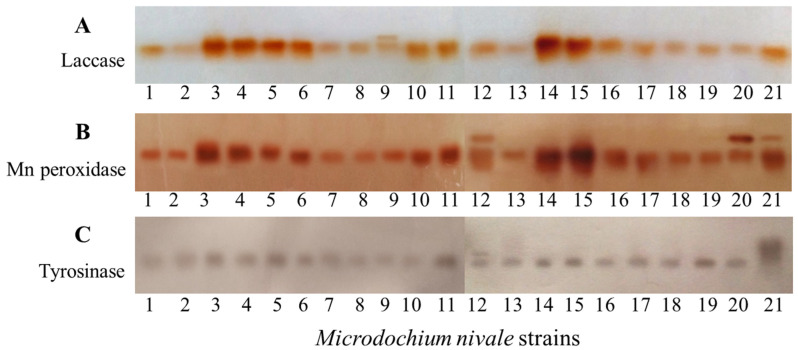
The staining of *Microdochium nivale* extracellular laccases (**A**), Mn peroxidases (**B**), and tyrosinases (**C**) by corresponding chromogenic substrates (o-dianisidine, 2,6-Dimethoxyphenol + MnSO_4_ + H_2_O_2_, and L-dihydroxyphenylalanine, respectively) after separation by native electrophoresis in a 7.5% polyacrylamide gel (PAAG). *M. nivale* strains were grown in potato sucrose broth medium for 20 days.

The patterns of extracellular MP, Lac, and Tyr accumulated in the cultural supernatants of *M. nivale* strains were analyzed by native gel-eletrophoresis and the subsequent staining of gels with chromogenic substrates of different enzymes. For most of the strains, the activities of extracellular MPs, Lac, and Tyr corresponded to a single band in a gel (Figure 5). However, this does not imply that these strains produced a single isoform of each enzyme because a single band can correspond to multiple isoforms. For some strains, two bands corresponding to Lac (strain 9) (Figure 5A), MP (strains 12, 20, and 21) (Figure 5B), and Tyr (strain 12) (Figure 5C) were revealed. This means that either the spectrum of isoforms or regulation of their production may vary in different *M. nivale* strains.

### 3.3. The Effect of Rye Extract on the Activities of POs of M. nivale

To check if the assayed enzymatic activities can be responsive to host plant metabolites, the effect of rye extract on the extracellular LP, MP, Lac, and Tyr activities of *M. nivale* was assessed. For these experiments, *M. nivale* strain 1, a highly virulent strain used as a model in our studies [60], was chosen. The strain was grown in MM for 10 days, and then 1/10 (*v*/*v*) of water extract of rye leaves or 1/10 (*v*/*v*) of water was added to the cultures. Enzymatic activities were measured at 12, 24, 48, 96, and 168 h after the addition of rye extract or water. No assayed enzymatic activities were detected in sterile rye extract.

**Figure 6:**
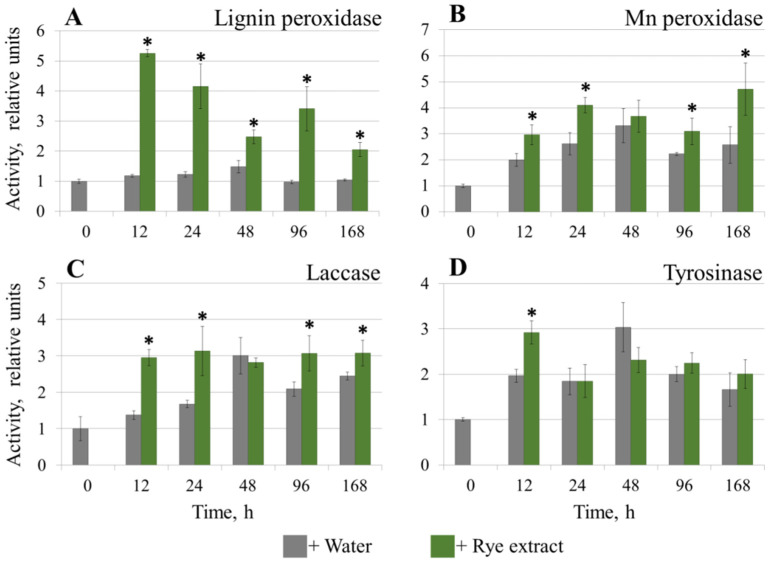
The effect of the extract of rye leaves on the extracellular activities of lignin peroxidase (**A**), Mn peroxidase (**B**), laccase (**C**), and tyrosinase (**D**) of the *Microdochium nivale* strain 1. Fungal cultures (grown for 20 days in minimal medium) were supplemented with rye extract (green columns) or water (gray columns), and then the enzymatic activities were measured. The values are given relative to the zero point (before the addition of rye extract or water), the activities at which were equated to one. The presented values are averages ± SD of five biological replicates. The stars show significant differences (Mann–Whitney U test, *p*-value < 0.05) between samples with and without rye extract.

The most pronounced induction by rye extract was observed for the extracellular LP activity in the *M. nivale* cultures (Figure 6A). The LP activity increased five-fold at 12 h after the addition of rye extract, and then decreased continuously, but remained significantly higher throughout the experiment than the LP activity in the *M. nivale* cultures incubated in the absence of rye extract. The MP and Lac activities were also higher in the presence of rye extract at four of the five assessed time points; however, the differences in the MP and Lac activities in the presence and absence of rye extract were rather small (less than two-fold) (Figure 6B,C). Tyr activity was the least responsive to rye extract, with no significant difference in the presence and absence of rye extract in four of the five time points tested, and the difference in one of the time points was only 48% (Figure 6D).

### 3.4. The Comparison of PO Activities with the Virulence of M. nivale Strains

To check whether the levels of the assayed extracellular PO activities corresponded to the level of virulence of *M. nivale* strains, a correlation analysis was performed. The virulence of the strains was assessed in our previous study based on the weight of the roots of infected plants (compared to non-infected plants) [50]. This parameter better reflects the virulence of *M. nivale* strains in quantitative terms compared to other morphometric parameters of infected plants [50]. In our present study, we did not find correlations between virulence (assessed by the host plant root weight) and any analyzed enzymatic activities at any time point or culture conditions, except in one case: the Lac activity under MM culture conditions after 30 days of cultivation had a negative correlation (Spearman correlation coefficient −0.726) with the virulence of the strains (Table S3, Supplementary Materials).

We proposed that a particular enzymatic activity might not correlate with virulence, but the combination of the activities might be important for virulence properties. To check this, we performed cluster analyses. Based on all assessed activities (under both culture conditions and at all analyzed time points), the assayed strains were clustered into three groups (described above, Figure 3), and each cluster contained both highly virulent and low virulent or avirulent strains. As such, the consideration of all activities together did not differentiate the strains according to their virulence properties. Therefore, we further considered the clusterization of the strains based on all combinations of three of four (LP + MP + Lac, LP + MP + Tyr, LP + Lac + Tyr, MP + Lac + Tyr) or two of four (LP + MP, LP + Lac, LP + Tyr, MP + Lac, MP + Tyr, Lac + Tyr) activities, both for two culture conditions together and for each condition separately. However, neither clusterization variant differentiated high- and low-virulent strains into separate clusters.

### 3.5. Extracellular Lignolytic Activity of M. nivale Strains

To check whether extracellular enzymes of *M. nivale* can decompose lignin and, if so, whether the extracellular enzymes of highly virulent strains have a more pronounced lignin-decomposing capacity compared to those of low virulent or avirulent strains, we first analyzed the cultural supernatants of six highly virulent strains. The supernatants of *M. nivale* cultures grown for 20 days in MM were added to the lignin-containing reaction mixture, and the dynamics of lignin degradation product accumulation was monitored spectrophotometrically for 20 min (one measurement per minute). To compare lignin decomposition intensity by the enzymes of different strains, the derivatives for the equations describing the curve of lignin degradation product accumulation were calculated. The values obtained for the extreme points (1 and 20 min) were substituted into the equations of the derivatives, and for the resulting values, the modules of differences were calculated to express lignin decomposition levels.

**Figure 7:**
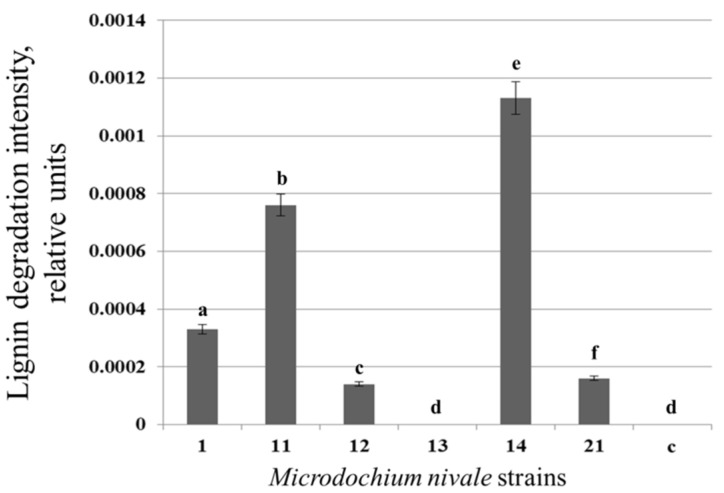
The intensity of lignin decomposition by the extracellular enzymes of highly virulent strains of *Microdochium nivale*. Fungal cultures were grown for 20 days in minimal medium, and then supernatants were collected and added to the reaction mixture with nitrated lignin. The accumulation of lignin degradation products was monitored spectrophotometrically. Lignin degradation intensity for each strain is expressed as a module of the difference between the values obtained from the derivative (calculated for equations of the curve of lignin degradation product accumulation) values of the function at extreme points. C—control sample without supernatants of fungal cultures. Columns that do not share the same letter have significant differences in pairwise comparison (Mann–Whitney, *p* < 0.05). The experiment was performed in four biological replicates.

Alterations in the optical density of the reaction mixture were observed only in the presence of hydrogen peroxide, indicating that peroxidases play a crucial role in lignin decomposition by *M. nivale*. The extracellular enzymes of the six analyzed highly virulent *M. nivale* strains differed in their lignin-decomposing capacity (Figure 7).

The supernatants of strain 13 did not cause lignin decomposition (Figure 7). For the supernatants of strain 11, we observed the highest lignin-decomposing capacity, which was 8, 7, 3, and 1.5 times higher than those of strains 12, 21, 1, and 11, respectively (Figure 7). The observed high variability (from null to high) in the lignin-decomposing capacity of the extracellular enzymes of six highly virulent *M. nivale* strains indicated that their high virulence could not be attributed to their high lignin-decomposing capacity; therefore, similar experiments have not been carried out for low-virulent or avirulent strains.

To check whether the lignin-decomposing capacity corresponded to the level of any assessed PO activity (measured after 20 days of incubation in MM medium), a correlation analysis was performed. A correlation was found between lignin-decomposition intensity and the level of Lac activity (Spearman correlation coefficient, 0.943). The activity levels of the other three types of enzymes did not correlate with lignin-decomposition intensity.

## 4. Discussion

The POs are poorly characterized in plant pathogens that cause diseases in non-woody plants despite the potential role of these enzymes in disease development. In our study, we assayed the extracellular activities of different POs (LP, MP, Lac, and Tyr) in the strains of the phytopathogenic fungus, *M. nivale*—a causal agent of different diseases of cereals and grasses [48,61,62]. We found that all 21 analyzed strains displayed all four enzymatic activities in vitro under the assayed conditions. Herewith, the analyzed strains, inhabiting a single crop (winter rye) in a single field, differed in the degree and dynamics of the assayed activities under both analyzed culture conditions. On average, in PSB, the medium with a rather rich content of growth substrate (30 g/L of sucrose) and a rather high diversity of sugars and other nutrients because of the presence of potato decoction, the levels of PO activities increased with the cultivation time. In contrast, in MM with low sugar content (3 g/L of glucose) and a poor diversity of carbon sources, the highest enzymatic activity levels, in many cases, were observed during earlier stages of fungal growth. This is in accordance with the induction of PO production at the reduced content/diversity of nutrients [8,59].

In terms of the pattern of PO activities, the assayed strains split into three clusters. The first cluster consolidated ten strains with relatively high extracellular PO enzymatic activities displayed in PSB and relatively low activities displayed in MM. The second cluster included three strains with rather high extracellular PO activities under both culture conditions. Within the third cluster, eight strains with rather low extracellular PO activities under both culture conditions were located.

We revealed that the co-regulation between MP and Lac activities, as well as between MP and Tyr activities, might occur in *M. nivale* strains, since correlations between these types of extracellular activities were found. In contrast, the LP activity did not correlate with any of the other three types of analyzed activities, indicating that the regulation of LP was probably carried out independently of MP, Lac, and Tyr activities. To the best of our knowledge, no correlation between the activities of different POs (and possible co-regulation of their production) has been previously analyzed in ascomycetes.

For most of the strains, we found that their extracellular MP, Lac, and Tyr corresponded to a single band in a gel after electrophoretic separation of the extracellular protein fraction, indicating that each strain produced either a single variant of each enzyme, or different variants with similar electrophoretic mobility. However, two bands, corresponding to the extracellular MP, Lac, and Tyr, were observed for some strains, suggesting that either the spectrum of isoforms or regulation of their production might vary in different *M. nivale* strains.

Among the assayed PO activities, the most responsive to the presence of host plant metabolites was LP: its activity increased five-fold after the addition of rye extract to the fungal culture. MP and Lac activities also increased in the presence of rye extract, but much less than the LP activity. The least responsive to rye extract among the analyzed extracellular activities was the Tyr activity. This likely means that different POs contribute to *M. nivale*–plant interactions to varying extents. Although the inducing effect of plant metabolites on the PO activities of basidiomycetes has been shown previously [59,63], as far as we know, this effect has not been demonstrated for the POs of ascomycetes before our study.

We did not find relationships between the virulence degrees of *M. nivale* strains and the levels of their extracellular PO activities. None of the enzymatic activities correlated positively with virulence at any assayed culture condition or at any time point. The clusterization of strains based on all four, three of four, and two of four enzymatic activities (in all possible combinations) did not split the analyzed strains according to their virulence level, irrespective of whether two culture conditions and three time points were analyzed together or separately. However, this does not mean that POs are unnecessary for *M. nivale*–plant interactions, especially given the potential role of these enzymes in lignin decomposition and the destruction of antimicrobial plant metabolites. It is possible that different *M. nivale* strains, having high phenotypic and genetic diversity and utilizing different strategies of interaction with the host [43,49,50], may rely on different POs during plant colonization, as well as in the course of other parts of their lifecycles, including the saprotrophic stages. Therefore, the correlations between PO activities and virulence may not be expressed. In our previous study, we showed that the activities of *M. nivale* extracellular enzymes, breaking glycosidic bonds, including various PCWDEs, also had no correlations with virulence [50]. Herewith, individual highly virulent strains vary greatly among each other in the activity of these enzymes. This likely means that each highly virulent *M. nivale* strain uses a unique “cocktail” of extracellular enzymes, and consequently applies a unique strategy of causing disease. Our present study shows that different POs are also likely to contribute to the “uniqueness” of the enzymatic cocktails that are delivered into host plant tissues by different strains of *M. nivale*.

The capacity of *M. nivale* extracellular enzymes to decompose lignin was also unassociated with the virulence of *M. nivale* strains. The extracellular enzymes of six highly virulent *M. nivale* strains differed in their lignin-decomposing capacity. For the extracellular enzymes of one of these strains, the lignin-decomposing activity was not detected. The enzymes of two strains were the “leaders” in terms of lignin degrading capacity, whereas one and two strains produced extracellular enzymes causing medium and low levels of lignin depolymerization, respectively. An unequivocal role in lignin decomposition by the *M. nivale* extracellular enzymes was played by peroxidases, since no lignin degradation products were detected in the absence of hydrogen peroxide in the reaction mixture. Whether it was LP or MP that played a dominant role in the lignin decomposition by *M. nivale* was not absolutely evident. All six analyzed strains possessed a rather high LP activity, which differed among these strains by only 2.6-fold. Therefore, the minor differences in LP activity among strains cannot account for the high variability in lignin decomposition. In turn, the MP activity differed among the analyzed strains by 17.5-fold. Moreover, the “strains-leaders” in lignin decomposition were characterized by the highest levels of MP, whereas the strain whose extracellular enzymes were unable to depolymerize lignin produced the least level of MP, indicating that MPs might determine the lignin decomposition capacity. However, we did not find a correlation between MP activity and the lignin decomposition level. The correlation with lignin decomposition level was revealed only for Lac activity, indicating that these enzymes are also likely to play a significant role in lignin depolymerization by *M. nivale*. Thus, the decomposition of lignin is probably a result of the coordinated action of different types of *M. nivale* POs, namely, LP, MP, and Lac.

The contribution of different POs to lignin degradation by different fungi remains debatable. In some studies, the crucial role in lignin decomposition was attributed to LPs and MPs, whereas Lac was considered an auxiliary enzyme in the degradation of this polymer [11,14,64,65]. The important role of MP in lignin decomposition was also supported by the fact that the strains displaying low levels of MP activity were deficient in their ability to decompose lignin, even if they produced high levels of LP [11,12]. This is in accordance with our data: *M. nivale* strain 13 possessed the lowest (among analyzed strains) level of extracellular MP activity, and its extracellular enzymes were unable to cause detectable lignin depolymerization; although, this strain possessed the highest (among analyzed strains) level of extracellular LP activity under assayed conditions. Other studies concluded an important role of Lac in lignin decomposition, since strains displaying a high level of lignin-degrading capacity had high Lac and MP activities, but did not obligatory exhibit LP activity [66]. The most active lignin destructors among the brown rot fungi also produced at least two types of lignolytic enzymes: MP and Lac [67,68]. In *M. nivale*, Lac was also likely to play an important role in lignin degradation, since we observed a correlation between the Lac activity in cultural supernatants and their lignin-decomposing capacity. Some authors are of the opinion that the lignolytic enzymatic complex is variable, and its composition is not universal in different species; here, species with different patterns of PO activities can cause similar levels of lignin decomposition [66,69].

Our study supports the hypothesis of the differential behavior of different closely related *M. nivale* strains, even if they inhabit a common crop in a common field. We suppose that the role of POs in plant–microbe interactions is highly underestimated not only for *M. nivale*, but also for other phytopathogens that cause diseases in non-woody plants. Given that the PO action may promote the destruction of the host plant tissues and plant defense metabolites, as well as the fact that the PO activities (at least some of them) can be induced by host plant extracts, these enzymes are obviously involved in the development of diseases, or at least contribute to pathogen fitness in planta.

## Data Availability

Not applicable.

## Supplementary Materials

The following supporting information can be downloaded at: https://www.mdpi.com/article/10.3390/jof8090918/s1, Table S1: Collection IDs of the *Microdochium nivale* strains used in this study; Table S2: Virulence of the *Microdochium nivale* strains used in this study; Table S3: The correlation analysis of the levels of extracellular phenol oxidase (lignin peroxidase, Mn peroxidase, laccase, and tyrosinase) activities and virulence of different *Microdochium nivale* strains.
