# Supplementary material for: Differential Activity of the Extracellular Phenoloxidases in Different Strains of the Phytopathogenic Fungus, Microdochium nivale
Source: J Fungi (Basel). 2022 Aug 29;8(9):918. doi: 10.3390/jof8090918 (PMC9502619; doi:10.3390/jof8090918)
Supplement: Supplementary file 1 [file jof-08-00918-s001.zip › Table S1 Strain IDs.pdf]

Table S1. Collection IDs of the *Microdochium nivale* strains used in this study.

Strain №	Strain ID in the collection
Microdochium nivale 1	F00608
Microdochium nivale 2	F00609
Microdochium nivale 3	F00610
Microdochium nivale 4	F00611
Microdochium nivale 5	F00612
Microdochium nivale 6	F00613
Microdochium nivale 7	F00614
Microdochium nivale 8	F00615
Microdochium nivale 9	F00616
Microdochium nivale 10	F00617
Microdochium nivale 11	F00618
Microdochium nivale 12	F00619
Microdochium nivale 13	F00620
Microdochium nivale 14	F00621
Microdochium nivale 15	F00622
Microdochium nivale 16	F00623
Microdochium nivale 17	F00624
Microdochium nivale 18	F00625
Microdochium nivale 19	F00626
Microdochium nivale 20	F00627
Microdochium nivale 21	F00628
