# Supplementary material for: Differential Activity of the Extracellular Phenoloxidases in Different Strains of the Phytopathogenic Fungus, Microdochium nivale
Source: J Fungi (Basel). 2022 Aug 29;8(9):918. doi: 10.3390/jof8090918 (PMC9502619; doi:10.3390/jof8090918)
Supplement: Supplementary file 1 [file jof-08-00918-s001.zip › Table S2 Virulence.pdf]

Table S2. Virulence of *Microdochium nivale* strains assessed based on the weight of the roots of infected plants (winter rye (*Secale cereale* L.) cultivar Ogonek) compared to non-infected plants (Gorshkov et al., 2020). The values show the percentage of root weight of infected plants compared to control. Highly virulent strains are marked by a yellow background.

Strain №	Percentage of root weight of infected plants compared to control, %
Microdochium nivale 1	18.6
Microdochium nivale 2	88.7
Microdochium nivale 3	61.9
Microdochium nivale 4	75.3
Microdochium nivale 5	19.5
Microdochium nivale 6	22.1
Microdochium nivale 7	55.0
Microdochium nivale 8	17.7
Microdochium nivale 9	17.7
Microdochium nivale 10	44.6
Microdochium nivale 11	14.3
Microdochium nivale 12	21.2
Microdochium nivale 13	26.4
Microdochium nivale 14	23.4
Microdochium nivale 15	24.7
Microdochium nivale 16	42.4
Microdochium nivale 17	70.6
Microdochium nivale 18	72.7
Microdochium nivale 19	75.8
Microdochium nivale 20	52.4
Microdochium nivale 21	11.7
